# Current Progress and Future Outlook for Synthetic Gene Circuits in Cardiovascular Therapy

**DOI:** 10.3390/biom16050754

**Published:** 2026-05-21

**Authors:** Mohammadali Khalilitousi, Arshaan Dhingra, Leili Rohani, Ron Weiss

**Affiliations:** 1 School of Biomedical Engineering, The University of British Columbia, Vancouver, BC V6T 1Z4, Canada; samtouss@mit.edu (M.K.); arshaan9@student.ubc.ca (A.D.); 2 Department of Biological Engineering, Massachusetts Institute of Technology, Cambridge, MA 02139, USA; leili_ro@mit.edu

**Keywords:** synthetic biology, cardiovascular diseases, synthetic gene circuits, cardiomyocytes, gene therapy, machine learning, gene regulatory networks, precision medicine

## Abstract

Despite decades of therapeutic advances, cardiovascular diseases remain the leading cause of global mortality, underscoring the need for strategies that move beyond untargeted systemic pharmacotherapy. Synthetic biology introduces a programmable therapeutic paradigm in which engineered gene circuits can sense, compute, and respond to pathological signals with spatiotemporal precision. This review examines the current progress of synthetic gene circuits for cardiovascular therapy, organized across three domains of clinical relevance. The first domain comprises circuits engineered for direct cardiac applications, from inducible switches to classifier systems. This discussion is further expanded by exploring circuits that indirectly target cardiovascular disease; these circuits address upstream risk factors such as cholesterol dysregulation and chronic inflammation. Looking ahead, the focus shifts to orthogonal architectures pioneered in other therapeutic contexts that hold promise for future cardiac applications. This review further discusses the emerging role of computational tools, including gene regulatory network inference and foundation models, in accelerating target discovery. Finally, a modified Design-Build-Test-Learn framework is proposed to overcome translational bottlenecks, thus paving the way for next-generation cardiac therapeutics.

## 1. Introduction

The biological understanding of cardiovascular development and disease has been constructed through a reductionist lens by isolating individual transcription factors (TFs) and signaling pathways to discern their functions [1,2,3,4]. This biological understanding has driven the development of the current standard of care, which ranges from delivery of small-molecule pharmacotherapies (e.g., beta-blockers and ACE inhibitors) to implantable electronic devices [5,6]. However, partly because these therapies act systemically and cannot adapt their dosing to fluctuating disease activity, cardiovascular diseases (CVDs) remain the leading cause of global morbidity and mortality [7]. Newer modalities, including AAV-mediated gene replacement, mRNA therapeutics, antisense oligonucleotides, and CRISPR-based genome editing, share the same fundamental limitation: each delivers a single payload that is expressed without spatial or temporal regulation, and their cardiovascular clinical translation has been correspondingly inconsistent [8]. Synthetic gene circuits (see Table 1 for glossary of terms) offer a compelling alternative paradigm by encoding therapeutic logic directly into genetic material [9]. This is particularly relevant in the heart, where mechanical load and signaling activity fluctuate across acute and chronic timescales and constitutive expression of therapeutic factors can itself drive maladaptive remodeling or arrhythmogenesis.

**Figure 1:**
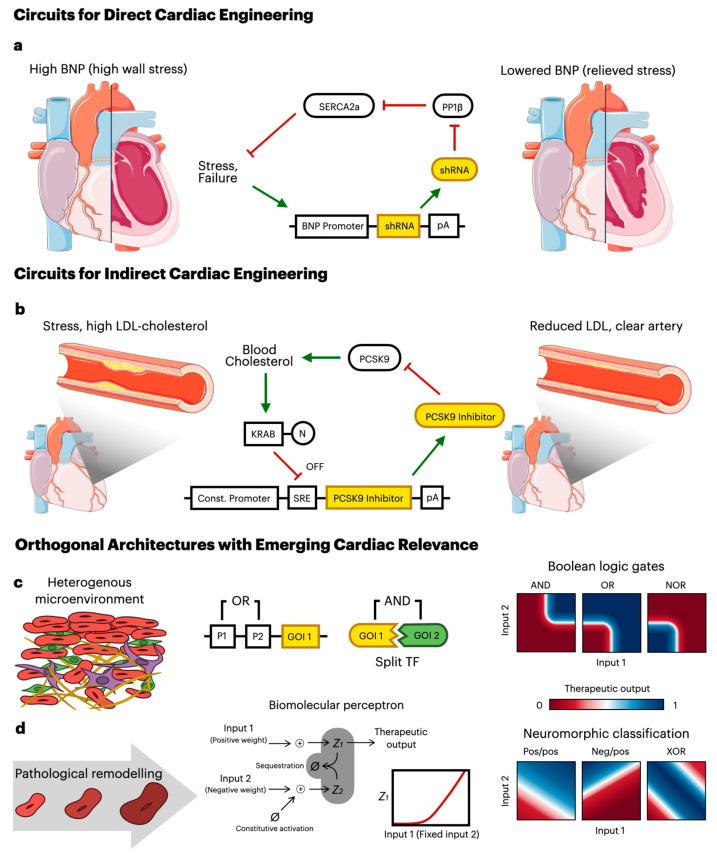
Illustrative examples of synthetic gene circuit architectures for cardiovascular applications (**a**) A closed-loop circuit for heart failure in which a *BNP* promoter, activated by cardiac wall stress, drives expression of a therapeutic shRNA that silences PP1β. This relieves PP1β-mediated suppression of SERCA2a, restoring Ca^2+^ handling. Since the therapeutic output alleviates the stress that drives promoter activation, the circuit self-regulates through host physiology (left: diseased state; right: relieved stress). (**b**) A closed-loop cholesterol-sensing circuit in which a constitutive promoter drives a PCSK9 inhibitor downstream of an SRE element. Under high cholesterol conditions, the KRAB–SREBP fusion protein is retained, permitting therapeutic expression. The resulting PCSK9 inhibition restores LDL receptor levels and reduces circulating cholesterol (left: atherosclerotic artery; right: cleared artery). (**c**) Examples of Boolean logic gates designed to classify cells within a heterogeneous cardiac microenvironment (left). An OR gate example that uses tandem promoters (P1/P2) to drive a single gene of interest, alongside an AND gate example that requires co-expression of split transcription factor components. Heatmaps (right) illustrate the resulting therapeutic output profiles for AND, OR, and NOR logic as a function of two varying inputs. (**d**) Biomolecular perceptron designed to process continuous analog signals associated with pathological remodeling (left, represented as a gradient of cardiomyocyte hypertrophy) for classification. Molecular sequestration between species Z_1_ and Z_2_ implements positive and negative computational weights, producing a non-linear activation function (inset). Heatmaps (right) show classification outputs for positive/positive, negative/positive, and XOR weight configurations, illustrating the capacity for complex non-linear decision-making.

This review explores the application of synthetic gene circuits in cardiovascular therapy across three domains. Firstly, circuits engineered for direct cardiac applications are discussed; these circuits range from externally inducible open-loop systems to implicitly closed-loop stress sensors and multi-input cell classifiers (Figure 1a). The discussion then extends to circuits that target CVD disease indirectly by sensing and correcting upstream systemic drivers such as hypercholesterolemia, metabolic dysfunction, and chronic inflammation (Figure 1b). Lastly, orthogonal circuit architectures that have been developed for other therapeutic contexts but hold significant promise for future cardiac applications are explored; these architectures include Boolean logic gates for classifying heterogeneous cell populations (Figure 1c) and neuromorphic biocomputers for analog decision-making along continuous disease gradients (Figure 1d). Although the presented circuit list is not exhaustive, it does highlight key developments that have shaped the field and will inform future therapeutic design strategies.

## 2. Landscape of Cardiovascular Circuit Applications

CVD emerges from various sources of dysfunction, with the primary source being a failing myocardium. Systemic drivers are also notable culprits of cardiac disease and include diseases like hypercholesterolemia, metabolic stress, and chronic inflammation. Current synthetic gene circuits offer the ability to address CVD by either acting directly on the myocardium or by targeting systemic drivers of the cardiac disease. However, more sophisticated applications require circuits that integrate multiple inputs to distinguish among related pathologies and tailor therapy to specific cell populations. Examples of such circuits have only been developed for applications extending beyond CVD. The following section examines circuits that directly and indirectly address CVD; more importantly, circuit architectures developed in adjacent therapeutic domains that have the potential to address the limitations of current circuits for CVD are introduced to expand the cardiac toolkit.

### 2.1. Circuits for Direct Cardiac Engineering

The simplest direct cardiac circuits are open-loop systems that rely on exogenous inducers to control therapeutic gene expression. Both Eriksson et al. [9] and Strobel et al. [10] focused on the tetracycline-dependent ribozyme K19, a riboswitch that destabilizes its host mRNA in the absence of ligand and stabilizes it upon tetracycline binding, providing reversible small-molecule control of protein output. Strobel et al. [10] demonstrated that the K19 switch enabled potent and reversible regulation across multiple organs in vivo. Eriksson et al. [9] further optimized the expression cassette for VEGF-B therapy by using dual riboswitches with a 100 bp linker, which reduced leakiness and improved dynamic range. Ligand-gated control addresses a known cardiac failure mode, since unregulated VEGF expression drives hemangioma-like lesions and pathological vessel growth in preclinical models [11]. Confining expression to defined therapeutic windows, as the tetracycline-gated cassette does, mitigates this risk.

An alternative exogenous trigger is light. Müller et al. [12] engineered a red-light–inducible split transcription factor circuit to drive expression of human vascular endothelial growth factor (hVEGF121). The system pairs a TetR–PIF6 fusion with a PhyB–VP16 fusion under a tetO-containing promoter; red-light illumination drives PhyB–PIF6 dimerization, reconstituting a functional transcriptional activator at the operator and switching VEGF expression on, while far-red light reverses the interaction. CHO-K1 cells expressing this module were embedded in a biocompatible hydrogel and applied to the chick chorioallantoic membrane; red-light illumination produced robust neovascularization with dense capillary networks, while far-red controls showed no such response. Although untested in cardiac tissue, this circuit’s spatially and temporally restricted angiogenic output is well-suited for ex vivo preconditioning of cardiac patches, engineered heart tissues, and epicardial cell sheets. In these contexts, tunable VEGF release could enhance graft vascularization and survival. However, direct in vivo myocardial deployment remains constrained by limited red-light penetration through the chest wall, motivating the development of upconversion nanoparticles and near-infrared–responsive optogenetic variants for transmural cardiac applications.

Moving beyond open-loop architectures, Miyazaki et al. [13] engineered a gene circuit to treat heart failure that functions as an implicitly closed-loop system; in this system, the feedback path runs through native cardiac physiology rather than through an engineered feedback node. In a failing myocardium, elevated protein phosphatase 1 (PP1) activity dephosphorylates phospholamban (PLN). This suppresses sarcoplasmic reticulum Ca^2+^-ATPase (SERCA2a) and impairs Ca^2+^ cycling, driving contractile dysfunction and electrical instability. The authors targeted PP1β, the highest-impact isoform, using a shRNA driven by a B-type natriuretic peptide (*BNP*) promoter, which is selectively activated in failing myocardium (Figure 1a). Upon promoter activation, the shRNA degraded *PP1β* mRNA, restoring PLN phosphorylation, SERCA2a-mediated Ca^2+^ reuptake, and overall calcium cycling. Critically, because the therapeutic output (restored Ca^2+^ handling) alleviates cardiac stress, the circuit self-regulates through the host’s own physiology without requiring a synthetic feedback component.

The most architecturally complex circuit with direct cardiac application is the cardiomyocyte-specific cell classifier developed by Magadum et al. [14]; this circuit uses multiple endogenous microRNA inputs to restrict therapeutic expression to cardiomyocytes. The authors engineered a modRNA delivery platform to express *Pkm2*, a glycolytic enzyme that regulates the cardiomyocyte cell cycle, using a two-component design: an *L7Ae* regulatory modRNA and a *Pkm2*-K modRNA. The *L7Ae* system, which consists of an RNA-binding protein that represses translation by binding to K-turn motifs, has been established in previous foundational studies for developing RNA-based cell type classifiers and broader RNA circuitry [15,16]. In this study’s design, the regulatory modRNA was tagged with microRNA response elements (MREs) recognizing cardiomyocyte-enriched miR-1 and miR-208a [17]. In cardiomyocytes, miRNA binding degrades the *L7Ae* transcript, leaving the K-turn–containing *Pkm2* modRNA free to translate; in non-cardiomyocytes, *L7Ae* is produced and represses *Pkm2* expression. Although Magadum et al. [14] framed this design as miRNA-mediated tissue-specific expression, it can be reconceptualized as an OR gate at the input layer; in this case, either cardiomyocyte miRNA can independently degrade *L7Ae* mRNA to de-repress therapeutic output. This represents a step toward the multi-input classifier architectures discussed in Section 2.3.

Beyond intracellular synthetic circuits, programmable engineered-cell therapies represent a complementary cardiac strategy. Aghajanian et al. [18] demonstrated that adoptively transferred CAR-T cells targeting fibroblast activation protein (FAP) reverse cardiac fibrosis in murine pressure-overload models; Rurik et al. [19] subsequently used LNP-delivered modified mRNA to generate transient anti-FAP CAR-T cells in vivo, avoiding permanent gene modification while restoring cardiac function. These adoptive-cell approaches fall outside the strict definition of intracellular logic circuits but illustrate the broader convergence of cardiac synthetic biology toward programmable cell-based therapeutics.

### 2.2. Circuits for Indirect Cardiac Engineering

CVD rarely arises in isolation. It is more often the downstream consequence of systemic risk factors such as hypercholesterolemia, metabolic dysfunction, and chronic inflammation that progressively damage the heart and vasculature. The following section therefore highlights circuits that target these upstream drivers, offering strategies with the potential to improve not only cardiovascular health but overall human health.

Unal et al. [20] engineered a synthetic gene circuit that repurposed the native cholesterol-sensing pathway to regulate endogenous cholesterol levels by controlling the expression of a therapeutic inhibitor of PCSK9, a protein that promotes degradation of the LDL receptor and thereby increases circulating cholesterol levels. Under high-cholesterol conditions, sterol regulatory element-binding proteins (SREBPs) are retained in their inactive form in the ER membrane through interaction with SCAP and INSIG-1; under low cholesterol, INSIG-1 dissociates from SCAP, permitting the SCAP–SREBP complex to traffic to the Golgi, where SREBP is cleaved to release N-terminal transcriptional activators that bind sterol regulatory elements (SREs) in the promoters of cholesterol biosynthesis and uptake genes [21]. To invert this intrinsic behavior, the authors fused the SREBP transcriptional domain to a KRAB repression module, producing a KRAB–SREBP fusion that retained cholesterol-dependent processing but functioned as a transcriptional repressor upon nuclear entry. Placing a PCSK9-inhibitory peptide (BMS-962476) downstream of an SRE-driven promoter inverted the relationship. Due to this, under low cholesterol conditions, the KRAB–SREBP fusion translocated to the nucleus and repressed peptide expression; on the other hand, under high cholesterol conditions the fusion remained ER-tethered and the peptide was expressed, inhibiting PCSK9 and lowering circulating LDL. As a single-input closed-loop system repurposing one endogenous sensing pathway, this circuit represents the simplest architecture in this section. Its cardiac relevance is direct, given that monoclonal anti-PCSK9 therapies (evolocumab, alirocumab) have demonstrated reductions in major adverse cardiovascular events in large outcomes trials [22]. A closed-loop genetic circuit producing endogenous PCSK9 inhibition could in principle deliver equivalent LDL-lowering with the dosing autonomy that bi-weekly injections cannot provide.

Adding a layer of complexity, Rössger et al. [23] engineered a closed-loop circuit for diet-induced obesity by coupling endogenous lipid sensing to an exogenous safety override. Under baseline conditions, the circuit maintained transgene expression in the “off” state; circulating free fatty acids activated PPAR-α through co-repressor release and co-activator recruitment, switching expression “on” to drive production of the appetite-suppressing hormone pramlintide, while a phloretin-responsive TtgR module provided independent exogenous control as a safety override. The inclusion of this dual-control architecture, combining endogenous metabolic sensing with an exogenous safety override, places this circuit at an intermediate level of complexity. Although obesity is the indication, the cardiovascular relevance is substantial since obesity drives hypertension, insulin resistance, and dyslipidemia [24]. The same architectural template (a metabolic sensor coupled to a clinician-controlled override) could be redirected to glucose-, leptin-, or lipid-dependent therapeutics for direct mitigation of cardiometabolic risk.

The most complex circuits presented here are designed to sense inflammatory biomarkers, which could be engineered to provide autonomous, localized therapeutic delivery for the treatment of atherosclerosis, myocardial infarction, and myocarditis. The CANTOS trial of canakinumab (anti–IL-1β) has provided proof-of-principle that targeting the IL-1/NF-κB axis reduces major adverse cardiovascular events independent of LDL lowering, motivating efforts to deliver such anti-inflammatory therapy with greater spatial and temporal precision than systemic monoclonal antibodies allow [25]. Smole et al. [26] used an NF-κB responsive promoter to detect pro-inflammatory cytokines and drive expression of the orthogonal transactivator Gal4-VP16 (GV16); a competing Gal4 DNA-binding domain occupied UAS sites at low GV16 levels, imposing a thresholding behavior that activated the circuit only once GV16 production exceeded a defined level, and the activated circuit then produced an anti-TNF-α antibody to suppress inflammation. This design directly addresses a known cardiac failure mode. Systemic anti-TNF therapy in heart failure (RECOVER, RENAISSANCE, ATTACH) failed clinically, in part because constitutive cytokine blockade disrupts homeostatic immune surveillance [27]. By contrast, an NF-κB–gated circuit releasing anti-TNF only when local inflammation crosses a threshold offers a plausible path around this limitation.

Cimino et al. [28] extended this inflammation-sensing approach by layering circadian regulation on top of NF-κB sensing. In their design, an NF-κB–responsive promoter detects pro-inflammatory cytokines while E′-box motifs couple expression to the endogenous BMAL1/CLOCK–PER/CRY oscillator. The result is rhythmic IL-1Ra release rather than continuous output. By layering circadian oscillation on top of inflammatory sensing, Cimino et al. constructed one of the most architecturally sophisticated closed-loop circuits applied to a cardiovascular-relevant pathology. IL-1Ra (anakinra) has direct cardiovascular precedent. VCU-ART showed favorable effects on left-ventricular remodeling post-myocardial infarction [29], and circadian gating is particularly attractive given that ischemic events themselves cluster in early-morning hours, suggesting natural alignment between circuit output timing and disease pathophysiology [30].

### 2.3. Circuit Architectures with Emerging Cardiac Relevance

Beyond the cardiovascular-specific circuits detailed in Section 2.1 and Section 2.2, a parallel body of work has produced powerful circuit architectures in other therapeutic domains, most notably oncology; the following architectures could serve as blueprints for future cardiac applications.

The simplest multi-input designs are built using Boolean logic gates. In cancer gene therapy, a dual-promoter circuit successfully implemented an AND gate for improved solid tumor-targeting precision [31]: expression of separate components of a split transcription factor required activation of two distinct tumor-specific promoters, with the reconstituted TF triggering target gene expression only when both inputs were detected. This dual-promoter design has been further enhanced and applied in identifying bladder cancer cells, suggesting a translatable architecture for addressing cardiovascular heterogeneity [32]. For instance, an analogous split-TF AND gate could combat cardiac fibrosis by requiring co-detection of a resident-fibroblast lineage marker (e.g., *TCF2* or *PDGFRA*) and a pathological activation marker (e.g., *POSTN* or *ACTA2*), confining therapeutic actuation to disease-driving myofibroblasts while sparing healthy resident fibroblasts and other mesenchymal cell types. Beyond dual-input systems, Angelici et al. [33] demonstrated AAV-based delivery for an AND gate between two TF inputs and up to six miRNA NOT-gate inputs in targeting multifocal tumors in the liver. By combining NOT logic across multiple miRNA inputs, this architecture effectively implements a NOR gate at the miRNA layer, suppressing therapeutic output if any off-target healthy-tissue miRNAs are detected and thereby providing a multiplexed safety filter. For cardiac applications, an analogous miRNA NOR layer using cardiac-restricted off-target sentinels could prevent therapeutic actuation in non-cardiac tissues following systemic AAV delivery, a known concern given AAV9’s broad tropism. Incorporating OR logic into these multi-input constructs further allows them to sense mutually redundant disease pathways, ensuring robust therapeutic actuation across heterogeneous pathogenic cell populations; engineers have achieved this compactly by combining tandem promoters with alternative splicing, such that any of several disease-activated promoters yields the same downstream therapeutic protein after splicing [34].

A complementary non-Boolean architecture is the synNotch receptor system, which uses contact-dependent proteolytic cleavage of an engineered transmembrane receptor to drive synthetic transcriptional programs in response to specific surface antigens [35,36]. Unlike the soluble-input logic gates above, synNotch requires direct cell–cell contact, which is precisely what makes it attractive for cardiac applications. Spatially restricted activation could target injured myocardial niches such as fibrotic scars or ischemic borderzones based on neighbor-cell identity rather than diffusible cues; this effectively addresses a known limitation of biomarker-driven circuits in zones where biomarker concentrations are spatially heterogeneous.

While Boolean logic circuits can provide powerful discrete control, physiological disease states in cardiology exist along continuous analog gradients. For example, circulating levels of established cardiovascular biomarkers such as NT-proBNP (N-terminal pro-B-type natriuretic peptide) and hs-cTn (high-sensitivity cardiac troponin) exhibit a continuous, dose-dependent relationship with the severity of heart failure, pathological remodeling, and subclinical myocardial injury [37,38]. Building circuits capable of responding proportionately to these gradients requires solving a more fundamental problem first: cell-to-cell variability in vector copy number produces baseline expression noise that can dominate over the biomarker signal the circuit is designed to sense. Recently developed dosage-compensating architectures, discussed in Section 3.2, enforce uniform expression independent of copy number, providing the stable baseline that analog computation requires.

Building upon current gene circuit foundations, the implementation of neuromorphic computation within living cells represents an emerging area of investigation. Moorman et al. [39] introduced the theoretical design concept of a Biomolecular Neural Network (BNN), a dynamic chemical reaction network intended to execute artificial neural network computations using molecular concentrations as continuous variables. At the core of a BNN is the biomolecular perceptron, which uses molecular sequestration to achieve negative computational weights and a non-linear activation function; assembling these perceptrons into multilayer feed-forward architectures would, in principle, allow cells to solve nonlinear classification tasks that single-layer circuits cannot, including canonical benchmarks such as the exclusive-OR (XOR) problem. In the context of CVD, where pathological remodeling involves subtle, continuous shifts across multiple overlapping gene regulatory networks, a BNN could in principle weight competing inputs to discriminate among related pathologies. For example, integrating *BNP*, troponin, and inflammatory cytokine concentrations could allow such a circuit to distinguish decompensated heart failure from acute infarction from myocarditis, and deploy a graded and condition-specific therapeutic response. To date, however, no in vivo BNN has been demonstrated in mammalian cells; clinical cardiac deployment therefore remains a long-term aspiration rather than a near-term prospect.

## 3. Clinical Translation Using a Data-Driven DBTL

**Figure 2:**
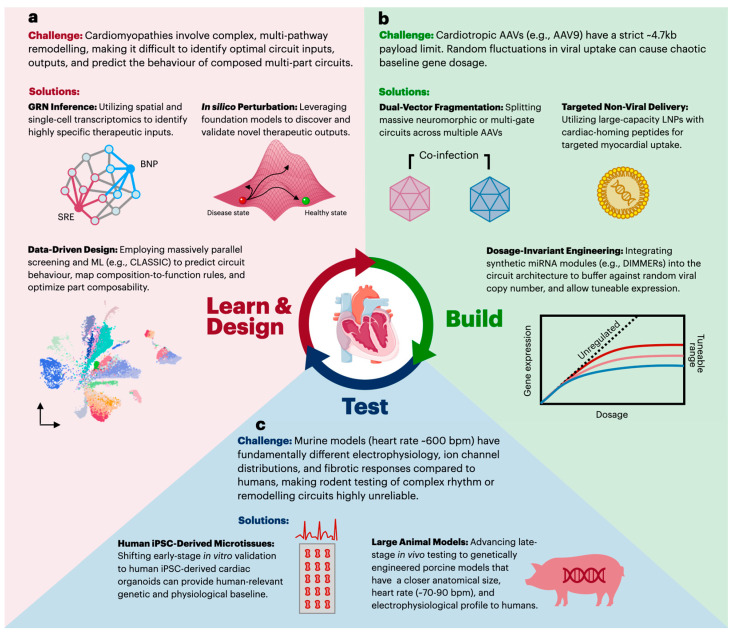
A proposed data-driven translational pipeline for cardiovascular synthetic biology. Adapting the traditional DBTL cycle, this three-node framework is tailored to cardiac applications, with examples at each node. (**a**) The complexity of multi-pathway cardiomyopathies can be approached by utilizing gene regulatory network (GRN) inference and foundation models for in silico target discovery, alongside high-throughput machine learning platforms (e.g., CLASSIC) to aid in circuit composability. (**b**) Viral packaging limits and baseline delivery noise can be addressed through dual-vector AAV fragmentation, targeted large-capacity lipid nanoparticles (LNPs), and the integration of dosage-compensating architectures (e.g., DIMMERs) can promote more uniform expression. (**c**) The interspecies physiological discordance between rodents and humans necessitates shifting early-stage in vitro validation toward human iPSC-derived microtissues and late-stage in vivo testing toward genetically engineered porcine models.

The clinical realization of complex cardiovascular genetic circuits is currently impeded by significant translational hurdles, most notably due to the limitations of in vivo delivery vehicles and the fundamental discordance between animal models and human cardiac physiology [40]. Therefore, clinical translation remains predominantly confined to single-gene designs, exemplified by the AAV1/SERCA2a replacement program in heart failure [41]. The translational pipeline for synthetic biology is commonly structured around a Design-Build-Test-Learn (DBTL) cycle, which integrates computational design, physical assembly, experimental validation, and iterative refinement [42]. However, as the complexity of circuits scales, this traditional four-step paradigm can prove inefficient: each iteration demands a full wet-lab cycle, and the sequential separation of “Design” from “Learn” prevents computational models from being updated as experimental data accrues. Advances in artificial intelligence and high-throughput screening are enabling a transition from stage-gated learning to continuous data assimilation, effectively consolidating the design cycle into three nodes. In this paradigm, “Design” and “Learn” merge into a unified data-driven computational hub that continuously utilizes empirical data to dynamically train machine learning models (Figure 2). These models not only aid in circuit design and simulation but also allow for the identification of novel cardiac therapeutic targets and circuit inputs.

Beyond translational hurdles, the circuits surveyed in Section 2 expose specific engineering limitations that motivate this framework. Cimino et al. [28] observed that inflammatory IL-1β signaling suppressed the output of circadian E′-box-driven circuits, illustrating how endogenous host pathway activity can distort intended circuit behavior; Smole et al. [26] deliberately selected the Gal4-VP16 transactivator for its orthogonality to mammalian signaling, while also noting that failing to dampen basal noise or restrict unwanted activation can compromise cardiac safety, where unintended variations in gene expression can disrupt cardiomyocytes and trigger life-threatening arrhythmias [43]. Metabolic load, meanwhile, remains largely unexamined in the cardiac gene circuit literature: studies like those of Miyazaki et al. [13], Unal et al. [20], and Eriksson et al. [9] focus predominantly on therapeutic output, dynamic range, and regulatory control while treating the producing cells as reliable expression platforms without assessing the metabolic cost borne by those cells. The proposed three-node cycle framework addresses these fundamental limitations by integrating metabolic cost and host-circuit interactions directly into the design and learning phases.

### 3.1. Data-Driven Design and Learning

The Learn and Design node of the modified DBTL cycle (Figure 2a) addresses a central challenge in cardiovascular circuit engineering: target identification and circuit design. Since CVDs range from static monogenic deficiencies to dynamic multi-pathway tissue remodeling, target selection and circuit design must be tailored to disease-specific pathophysiology [44]. Advanced cardiomyopathies and heart failure involve complex remodeling; thus, designing analog or multi-input circuits to accurately process these heterogeneous biological signals is exceptionally challenging. This is primarily because of the inherent unpredictability of how multiple genetic parts behave when composed into multi-kilobase networks. Addressing this challenge requires a dual-level computational approach: the first level identifies optimal molecular inputs and outputs for circuit encoding, while the second predicts the dynamic performance of the circuit within the intracellular environment.

In the first phase, computational models can accelerate target discovery by filtering vast transcriptomic datasets into high-confidence candidate sensors and actuators [45,46,47,48]. By identifying critical TFs, cis-regulatory elements, and highly specific promoter activity in pathogenic cell states, GRN modeling enables the selection of ideal promoters for spatial specificity, biomarkers for environmental sensing, and combinatorial markers for complex state classification. GRN inference frameworks like SCENIC move beyond simple correlation by identifying motif-supported and TF-driven regulons; recent studies using SCENIC have revealed differential activity of key regulators like the *HOX* family, *PPARA*, and *NR2F2* in specific cardiomyocyte clusters [48,49,50]. The activation of these TFs in various cell states could provide early-sensing inputs for synthetic promoters and, allowing circuits to respond to the cell’s precise metabolic and maturation state rather than late-stage structural markers. While SCENIC excels at identifying candidate circuit inputs through regulatory mapping, transformer-based foundation models like Geneformer are now being utilized to discover novel circuit outputs through in silico perturbation [46]. Geneformer was used to predict that the combinatorial knockout of *GSN* and *PLN* would shift the transcriptomic signature of dilated cardiomyopathy (DCM) toward a healthy state; these predictions were subsequently tested in iPSC-derived microtissues, where genetic knockouts improved contractile function. Despite such successes, the clinical utility of these foundation models remains tethered to the availability of massive training corpora. Furthermore, predictive accuracy often degrades when models are applied to rare or underrepresented cardiac subpopulations. Beyond these robustness concerns, recent benchmarking studies have raised serious doubts regarding transformer-based models’ capability to outperform simpler baselines on key cellular tasks; overall, the key takeaway from these studies is the necessity to perform rigorous application-specific validation, instead of blind adoption [51]. Nevertheless, by acting as efficient computational sieves, such tools may help reduce years of trial-and-error target identification.

Once a candidate list of molecular inputs and outputs is established, the engineering challenge shifts to the second level: defining and simulating the circuit’s dynamic behavior. Biological environments are inherently noisy and stochastic; consequently, a circuit that appears robust in silico (especially in deterministic models) may fail in vivo due to unforeseen timing mismatches, metabolic burden, or leakiness (unintended expression) in a cardiomyocyte [52,53]. Furthermore, even perfect simulation at the single-cell level cannot fully capture emergent properties at the cardiac tissue scale, which underscores the necessity of iterative wet-lab validation to bridge the gap between computational prediction and multicellular reality.

Deterministic modeling of circuit behavior using ordinary differential equations (ODEs) remains the standard for simulating circuit kinetics, especially for circuits displaying nonlinear dynamics such as oscillations or bistability. In brief, the standard workflow translates a qualitative interaction graph, representing activation and repression relationships between circuit components, into a system of differential equations describing time-varying molecular concentrations [54,55]. By analyzing the resulting parameter space (e.g., transcription rates, binding affinities), researchers can identify which specific genetic parts require tuning to achieve a desired output. However, standard ODE models assume a well-mixed environment, which is often insufficient for cardiac tissues characterized by complex geometry and cell–cell communication. To address this, ODEs are increasingly coupled with partial differential equations (PDEs) or agent-based models (ABMs) to simulate spatiotemporal dynamics, such as the diffusion of paracrine signals across 3D cardiac microtissues [56] (with stochastic approaches such as the Gillespie algorithm providing a complementary framework for low-copy-number regimes where intrinsic noise becomes non-negligible).

Biophysical models can provide mechanistic insight but often struggle to accurately predict the behavior of complex, multi-part circuits due to unknown context-dependent interactions. High-throughput experimental platforms can complement them by providing large-scale empirical datasets needed to train predictive machine learning models. An example of a high-throughput screening platform is CLASSIC (Combining Long- and Short-range Sequencing to Investigate Genetic Complexity), which allows users to physically construct and test over 10^5^ circuit design variants in a single experiment [57]. By mapping the entire design landscape, CLASSIC revealed non-intuitive composability rules; for instance, it identified that optimal AND-gate logic often required specific combinations of medium-affinity parts rather than the strongest available components.

### 3.2. Build and Delivery

The choice of delivery vehicle is often the first design decision in any cardiac circuit, as it sets hard constraints on payload size, cell-type tropism, and the feasibility of repeat dosing; yet delivery of synthetic circuits to the human myocardium remains a bottleneck (Figure 2b). While cardiotropic adeno-associated viruses (e.g., AAV9) remain the gold standard for cardiac transduction, their restricted packaging capacity (~4.7 kb) imposes significant constraints on the use of complex circuit architectures. [58,59]. For context, key cardiac payloads such as SERCA2a (~3.0 kb CDS) [60] and MYBPC3 (~3.8 kb CDS) [61] individually approach the AAV ceiling; therefore, any multi-input cardiac circuit that combines a cardiac-specific promoter, sensor module, and therapeutic effector would exceed the single-vector capacity. Furthermore, random fluctuations in viral uptake in vivo cause chaotic baseline gene dosages, generating delivery noise that drowns out the circuit’s intended biological computation [62,63]. The immunogenicity of bacterial-derived payloads (e.g., Cas9, TetR) also poses a significant risk of cytotoxic T-cell responses and myocarditis [64,65]. Recent fatalities in high-dose systemic AAV trials have further intensified scrutiny of capsid-mediated immunogenicity and dose-dependent toxicity. These vulnerabilities are especially critical for cardiovascular therapies, which may necessitate repeat dosing, thereby increasing the risk of adverse immunological responses. [66].

To overcome the restrictive packaging capacity of AAV vectors, dual-vector systems can be used for modular splitting of expansive circuits across multiple viral particles [67,68]. Upon co-infection of a cardiomyocyte, components delivered on separate vectors can be reconstituted at the DNA level (e.g., trans-splicing or homologous recombination of paired ITRs to reconstruct a single transgene), the RNA level (e.g., split intron-mediated mRNA trans-splicing), or the protein level (e.g., split inteins for post-translational protein splicing, or split transactivators that reassemble functional regulators) [69]. However, this dual-AAV approach is heavily constrained by the statistical probability of co-infection, thus requiring significantly higher total viral doses to be administered. This can exacerbate the risk of hepatotoxicity, vector-induced inflammation, and amplification of the host immune response against both the viral capsids and the synthetic payload [66,70].

To circumvent viral payload constraints, non-viral platforms such as large-capacity lipid nanoparticles (LNPs) engineered with cardiac-homing peptides could be investigated for targeted myocardial uptake [71,72,73]. Yet, regardless of the chosen delivery vehicle, mitigating the challenge of chaotic baseline gene dosage remains critical; to address this, several dosage-compensating architectures have recently been developed that integrate directly into the circuit design [74,75]. A prominent example is the DIMMER (Dosage Invariant miRNA-Mediated Expression Regulator) platform introduced by Du et al. [76], which embeds synthetic miRNA modules into the network to buffer against random vector copy numbers. By exploiting multivalent TNRC6-mediated mRNA regulation, these circuits achieved tunable and uniform expression profiles across roughly two orders of magnitude in gene dosage, thereby making them ideal for analog and multi-input computation.

### 3.3. Test and Validation

Testing complex genetic circuits for CVD faces profound physiological and regulatory hurdles (Figure 2c). First and foremost, there are severe interspecies physiological discordances; for example, small rodent models exhibit a heart rate of 300–400 bpm (vs. 60–70 bpm in humans) at rest and possess fundamentally different electrophysiology, ion channel distributions, and fibrotic responses compared to humans, thus making rodent models an inaccurate testing platform [77,78]. Second, in the electrically coupled syncytium of the heart, safety margins are exceedingly narrow. Unlike solid tumors, where off-target toxicity is localized, leaky expression in the heart could alter ion channel dynamics and trigger fatal arrhythmias [43]. This danger is compounded by the autonomous nature of closed-loop systems; if a sensor malfunctions or physiology shifts, the circuit could over-actuate without physician oversight [79]; consequently, high fidelity validation requires shifting the “Test” phase toward human-relevant cardiac physiology, supported by rigorous safety and regulatory frameworks. The empirical data generated from this phase can then directly feed back into the “Learn/Design” stage of circuit design.

Early-stage in vitro validation is shifting heavily toward human induced pluripotent stem cell (iPSC)-derived cardiac organoids and organ-on-a-chip platforms [80,81]. By providing a human-relevant genetic and physiological baseline, these platforms allow for high-throughput iteration, validation, and optimization of circuit performance and electrophysiological safety. However, this approach is fundamentally limited as iPSC-derived cardiomyocytes often retain immature metabolic and structural phenotypes [82]. Although emerging maturation protocols (e.g., metabolic conditioning and electromechanical training) show promise, whether they can sufficiently close this gap for high-fidelity circuit validation remains contested; this is partly because these isolated micro-platforms still lack the full neurohormonal, hemodynamic, and immunological complexity of a living patient [83].

To bridge this systemic gap, late-stage in vivo testing is advancing toward genetically engineered larger animal models [77]. Most notable among these recent developments are engineered porcine models [84]. Unlike rodents, pigs are closer in anatomical size, baseline heart rate, and electrophysiological profile to humans; thus, pigs offer a more predictive translational steppingstone for assessing vector biodistribution, circuit safety, and therapeutic efficacy prior to clinical trials. Unfortunately, the use of large animal models is limited due to prohibitive costs, low throughput, ethical constraints, and technical difficulty with generating accurate multi-genic disease phenotypes [85]. By improving current testing protocols, gene circuits could be safe enough to seamlessly integrate into clinical workflows. In this case, a patient’s cells would be reprogrammed into iPSC-derived microtissues for initial circuit calibration, then validated in genetically matched porcine models before delivery. Once administered, the circuit’s closed-loop architecture would provide patient-specific adaptability, autonomously scaling its therapeutic output to match the individual’s real-time physiological fluctuations.

## 4. Conclusions and Outlook

The application of synthetic gene circuits to cardiovascular medicine represents a shift from static symptom management to programmable interventions tailored to the cellular microenvironment. The current landscape of cardiovascular circuit engineering spans three domains of clinical relevance. Circuits with direct cardiac applications have demonstrated targeted therapeutic actuation within the myocardium. In contrast, circuits addressing upstream systemic drivers of CVDs have illustrated the capacity to treat pathology beyond the myocardium, with cholesterol-sensing, metabolic, and anti-inflammatory architectures autonomously correcting the pathological milieu that damages the heart over time. Orthogonal architectures (e.g., Boolean logic gates, dosage-compensating regulators, and biomolecular neural networks) pioneered in oncology and other domains provide a blueprint for the multi-input precision that cardiac tissue heterogeneity demands.

Underpinning the design of all these circuits is the emerging role of computational target discovery. GRN inference frameworks and transformer-based foundation models are examples of high-throughput methods that supply candidate molecular inputs and outputs for synthetic circuits. As these computational tools become more accurate, particularly for rare and underrepresented cardiac subpopulations, they will become increasingly integral to the earliest stages of circuit design.

The future of synthetic gene circuits hinges on bridging the gap between theoretical circuit design and clinical relevance and efficacy. By leveraging machine learning and high-throughput platforms like CLASSIC for in silico circuit optimization, advanced cardiotropic vectors and miRNA modules to bypass delivery and dosage constraints, and human iPSC-derived microtissues or large animal models for rigorous electrophysiological safety validation, bioengineers could overcome current translational bottlenecks. However, several areas warrant further optimization. First, the physical implementation and long-term maintenance of analog biomolecular neural networks in mammalian systems must be tested further. Second, regulatory frameworks need to be developed to accommodate autonomous therapeutics. Third, strategies to extend circuit persistence are needed to match the chronic nature of CVD. Finally, further work is required to address the spatial heterogeneity of the injured myocardium. If these challenges can be addressed, synthetic gene circuits have the potential to move beyond proof-of-concept demonstrations and become a genuinely transformative complement to the current standard of cardiovascular care.

## Figures and Tables

**Table 1:** Glossary of terms.

Term	Definition
**Genetic circuit**	an engineered biological network constructed from modular components that can sense inputs, generate outputs, or process signals to perform dynamic functions in varying contexts
**Open-loop circuit**	a circuit that executes a pre-programmed output in response to an exogenous inducer or spatial cue, without autonomous feedback from either the disease state or the circuit itself
**Closed-loop circuit**	a circuit that autonomously senses endogenous disease proxies and dynamically adjusts its therapeutic output, whether the feedback path runs through host physiology (implicitly closed-loop) or through an engineered circuit component (explicitly closed-loop)
**Multi-input circuit**	a circuit that integrates two or more independent signals through Boolean logic or analog computation; multi-input architectures can be either open-loop or closed-loop
**AND gate**	a logic architecture requiring the simultaneous detection of two distinct condition-specific promoters (e.g., a fibroblast-specific promoter combined with a pathological activation marker).
**OR gate**	a logic architecture that allows circuits to sense mutually redundant disease pathways, enabling therapeutic actuation across heterogeneous pathogenic cell populations
**NOT gate**	a logic architecture that suppresses expression in the presence of a specific input; often used as a safety mechanism to spare healthy, non-target cells by silencing output when healthy markers are detected
**Gene regulatory network (GRN)**	a network of regulatory interactions between transcription factors and their target genes; GRNs can be inferred from high-throughput data (e.g., single-cell transcriptomics) to identify critical transcription factors, cis-regulatory elements, and specific promoter activity
**Transformer-based foundation model (e.g., Geneformer)**	a predictive large-scale AI model pre-trained on vast, diverse, and often unlabeled datasets (biological in our case) that can be used to discover therapeutic targets through in silico perturbation, among other uses
**Ordinary differential equation (ODE)**	a type of mathematical equation describing the rate of change in a variable over time; systems of ODEs are used to model the kinetics and nonlinear dynamics (e.g., oscillations, bistability) of genetic circuits, assuming a well-mixed environment

## Data Availability

No new data were created or analyzed in this study.
